# Mediterranean Pattern Diet in Multiple Sclerosis: A Review Focusing on Immunometabolites

**DOI:** 10.2174/011570159X382929250719084728

**Published:** 2025-08-07

**Authors:** Giulio Papiri, Cristina Paci, Giordano D’Andreamatteo, Valentina Membrino, Tiziana Di Crescenzo, Gabriella Cacchiò, Claudia Cagnetti, Arianna Vignini

**Affiliations:** 1 Neurology Unit, Ospedale Provinciale "Madonna del Soccorso", 63074 San Benedetto del Tronto, Italy;; 2 Department of Clinical Sciences, Section of Biochemistry, Biology and Physics, Università Politecnica delle Marche, 60100, Ancona, Italy

**Keywords:** Multiple sclerosis, central nervous system, diet, immunometabolites, mediterranean pattern diet, MS pathology

## Abstract

Multiple Sclerosis (MS), the most common demyelinating disease of the Central Nervous System (CNS), is characterized in its pathogenesis by an interplay of mechanisms pertaining to aberrant immune response, acute and chronic inflammation, glial housekeeping, and neuron survival, ultimately resulting in demyelination, synaptic dysfunction, and neuroaxonal loss. Experimental models as well as epidemiological observations support the hypothesis of a role of diet in the disease onset, activity, and progression. It has been suggested that Western-type diets might be detrimental, while on the other hand, certain dietary regimens, like Mediterranean, low-fat, ketogenic, or intermittent fasting, might lead to disease amelioration, possibly through differential regulatory effects upon inflammation, immunity, and regenerative processes of neurons and glia. Under this perspective, immunometabolites, small intermediates including among the others citrate, itaconate, lactate, glutamate, glutamine, alfa-ketoglutarate, 2-hydroxyglutarate, fumarate, ceramides, whose turn-over reflects metabolic reprogramming of immune cells, might be viewed as significant regulators of cellular responses against either local or systemic noxious stimuli, both in the periphery and in the CNS. The present narrative review aims at summarizing current experimental and clinical evidence regarding the role of immunometabolites in shaping MS pathology, to address whether they could be relevant either as disease markers or therapeutic targets, and whether they might be differentially influenced by dietary approaches, especially by Mediterranean Pattern Diets (MPD).

## 1 INTRODUCTION

### 1.1 MS Aetiology and Pathogenesis

Multiple Sclerosis (MS) represents the prototypical demyelinating disease of the Central Nervous System (CNS), as well as the most common, constituting a debilitating disorder regarded as the most frequent cause of motor disability in young adults [1]. Demyelination might affect all anatomical structures of the CNS, preferentially the optic nerve, sensorimotor, coordination, and visual pathways; higher lesion load correlates with disability and death [2, 3]. Despite substantial continuity in the clinical course of MS, three main patterns are commonly identified: Relapsing Remitting forms (RRMS), characterized by recurring episodes of neurologic dysfunction - called relapses - followed by partial or complete recovery; Progressive forms (PMS), which may be primary (starting from disease onset) or secondary (evolving from RRMS), and is marked by sustained disability progression independent of relapses or lesion load increase; and finally, benign forms, characterized by prolonged clinical and radiological stability [4]. The aetiology of the disease is still unknown and is thought to be multifactorial, originating from an interaction between genetic predisposition and various environmental stimuli. Among acquired risk factors, viral infections, low sunlight exposure, and low plasma vitamin D levels, smoking, as well as obesity and a high-salt diet, have been associated with an increased likelihood of developing the disease [5]. Western countries show higher prevalence, whereas infancy, adolescence, and young adulthood appear critical for disease development, since people emigrated from low-incidence to high-incidence countries in their infancy or adolescence appear to acquire a risk of developing the disease similar to native dwellers [6]. Disease onset is ultimately thought to stem from the interaction of genetic, epigenetic, and environmental factors. The role of genetic predisposition has been demonstrated by the increased odds of MS in twins or first-degree relatives; on the other hand, variants of the Human Leukocyte Antigen (HLA) class 2 loci have been found to be associated with disease onset. HLADRB1*15:01 is considered the genetic trait more strongly associated with increased MS susceptibility, while HLA-A*02 has been associated with a protective role [7]. Epigenetic variations might significantly interact with genetic make-up: a recent Mendelian randomization study has suggested that HLA DRB1 methylation status mediates the effects of HLADRB1*15:01 [8].

MS pathogenesis builds upon the work of several cellular elements, including immune cells, neurons, glial elements, and endothelial cells, mainly within the framework of inflammatory cell migration from the periphery and related responses within the CNS [9, 10]. Apart from the role of inflammation in sustaining demyelination, several processes of glial cells, neurons and vascular elements, have been found in experimental models to concur to the disease; among these processes, growth factor signaling, mitochondrial genesis/autophagy, oxidative stress/peroxidation, synaptic remodeling, perturbed calcium homeostasis, myelin synthesis, endothelial dysfunction, neurovascular coupling and programmed cell death pathways are thought to play a significant role [9, 11]. In addition, processes tied to autoimmunity and demyelination might hasten CNS biological aging, converging on the so-called “inflammaging”. In keeping with this hypothesis, in MS, an increase in cellular aging markers, such as telomere shortening, has been reported [12]. MS is therefore considered a disease where CNS-autonomous pathways intertwine with aberrant immune activity, thus resulting in variable degrees of neuroaxonal loss, counterbalanced by CNS repair attempts, termed as regenerative plasticity, which involves remyelination, neurogenesis, and synaptic plasticity/restoration. To sum up, MS pathogenesis is thought to derive from the interplay of inflammatory processes, neuroaxonal loss, which involves autonomous processes of neurons and glial cells, *in situ* remyelination, and regenerative plasticity of synapses and high-order connections, which allows to preserve local and distant network integrity [13, 14]. During all phases of the disease, immunologic mechanisms do play a pivotal role. Several lines of evidence from experimental models, as well as the therapeutic effectiveness of immunomodulator drugs, point to a crucial role of acute and chronic inflammation, which undergoes a complex systemic and CNS-compartmentalized regulation in MS pathology [15]. In particular, MS has been mainly considered a T-cell sustained autoimmune disease, having at its core aberrant activity of CD4^+^ subtypes, like T helper type 1 (Th1), T helper type 2 (Th2), T helper type 9 (Th9), T helper type 17 (Th17) and cytotoxic CD8^+^ T cells [16]. In the last two decades, the role of B cells, plasma cells, and their innate immunity effectors, such as autoreactive macrophages and Natural Killer (NK) cells, has been increasingly recognized. Classical mechanisms of autoimmune diseases, like bystander activation, epitope spreading, and loss of self-directed tolerance, have been demonstrated to occur within the disease. In addition to effector lymphocytes, other subpopulations, such as T regulatory (Treg) cells, M2 macrophages, and mastocytes, have been recognized as negative regulators of the inflammatory response [17, 18], constituting a continuous spectrum of immune cells with intermediate phenotypes.

Among systemic factors, gut dysbiosis, through gut permeability, lipopolysaccharide, antigen, and antibody translocation as well as autoreactive cell differentiation, is thought to contrast peripheral immune tolerance and increase autoimmune responses [19]. Other proinflammatory systemic factors, like sex-hormone imbalance, sodium retention, and proinflammatory vasoactive peptides, like endothelins and angiotensin II, might play a role. At present, despite recent breakthroughs in immunomodulatory therapies, with the introduction of highly effective drugs targeting the B-cell axis, such as ocrelizumab or Bruton's tyrosine kinase (BTK) inhibitors, drugs targeting neuroaxonal loss or mitochondrial dysfunction are not available [15, 20]. Considering these pathogenetic aspects of MS, several lines of evidence point to the fact energy metabolism appears to be a reasonable target against the neurodegenerative component of the disease.

### 1.2 MS Pathogenesis, Mechanisms of Neuroaxonal Loss

In MS, autoimmunity and neurodegenerative processes are thought to be partly independent, interlocking from presymptomatic phases. Evidence from preclinical studies suggests that inflammation triggers harmful phenotypical changes in the CNS, affecting communication between neurons and glial cells. In particular, glial elements react to inflammatory stimuli by skewing differentiation towards a spectrum of reactive proinflammatory and neurotoxic phenotypes, which in simplified terms might be summarized as M1-polarized microglial cells [21], A1-polarized astrocytes [22], and immunoreactive oligodendrocytes [23, 24]. These cells, through inflammatory signaling, promote in the shortterm a protective response characterized by the removal of pathogenic stimuli and damaged structures. In contrast, their unrestricted activation might increase oxidative stress and disruption of energetic metabolism, ultimately leading to synaptic dysfunction and activation of cell death cascades in neurons, such as apoptosis and ferroptosis [25]. Reactive glia within demyelinating lesions rewires metabolism to promote inflammation, resulting in a positive feedback loop with augmented proinflammatory cytokine production and blood- brain barrier (BBB) permeabilization. These processes also characterize the so-called “slowly evolving lesions”, typical of progressive MS, which are defined by a low degree of inflammation, abundant activated microglia/macrophages and proliferating oligodendrocytes at the external borders, combined with histopathological signs of neuronal structural damage [26-28]. In addition to structural changes, production of circulating mediators like growth factors, neurosteroids, and neurotransmitters is affected [29, 30], ultimately contrasting remyelination [31]. As for neurotransmission, local imbalances in glutamate and GABA homeostasis, in addition to their neuronal effects, might upregulate effector functions of macrophages and T lymphocytes [32-34]. Neurons within demyelinating foci, which are stressed by increased metabolic demands, cytokine/neurotransmitter imbalance, and oxidative stress, might progress towards mitochondrial dysfunction, calcium overload, endoplasmic reticulum stress, and ultimately cell death [15, 20, 35]. Neuroaxonal loss is not thought to be an exclusive feature of demyelinating lesions, since normal appearing gray and white matter show subtle neuropathologic signs, characterized by structural synaptic changes, glymphatic outflow, and BBB disruption, together with impaired neurovascular coupling [36, 37]. Metabolic alterations also occur within non-demyelinated areas. Brier and coll have observed increased glycolysis and reduced oxygen utilization in normal-appearing areas of the CNS in MS patients, especially in subjects with longer disease duration [38]. Ultimately, the end-stage cellular responses underlying neuroaxonal loss are thought to share significant commonalities with the pathogenesis of ischemic damage, especially regarding the interplay between calcium signaling, endoplasmic reticulum stress, mitochondrial dysfunction, and programmed cell death [39, 40]. These “CNS-autonomous” pathogenetic processes are thought to some extent to be a common ground with other neurodegenerative diseases [41, 42], where energy failure is a central pathway during early and late phases [43-45]. Therefore, MS might not precisely fit under the classification of a fully autoimmune disease, even *ab initio* or as a *“two-stage”* disease, where neurodegenerative changes are merely the consequence of chronic inflammation [26]. In keeping with this, in recent years animal models such as Experimental Autoimmune Encephalitis (EAE) and toxin-induced demyelination models have confirmed a significant involvement of mitochondrial turnover and synaptic/energy failure in MS [40, 46, 47]. It has been shown that neurons might reconfigure their metabolism when ATP production is impaired, in order to preserve energy balance and mitochondrial function [48]. Furthermore, post-mortem MS studies have confirmed within lesions altered expression of electron transport chain proteins, especially Complex I and III [49]. Interestingly, long-lasting metabolic profile changes also occur in immune cells involved in chronic inflammation and autoimmunity. A recent study from Armon-Omer *et al*. has shown that circulating lymphocytes from MS patients display a degree of mitochondrial dysfunction which correlates with disease severity, suggesting that this aspect might be a valid shared target in disease pathogenesis [50]. Fransson *et al*. have observed that macrophages from MS patients display a persistent proinflammatory gene expression profile characterized by upregulation of glycolytic genes and downregulation of mitochondrial oxidative metabolism [51]. Therefore, research has tried to address whether cycling between different metabolic programs might occur through shared switches in both immune and nerve cells in MS.

### 1.3 Metabolic Reprogramming

The tight relationship between energy metabolism and oxidative stress in immune cells and neurons, which augments energy expenditure during periods of intense activity, suggests that metabolic adaptation of both cell types to conditions of chronic stress might be pivotal, respectively, during inflammation and after myelin damage. Therefore, in MS, it has been suggested that autoimmune responses as well as dysfunction of the glio-neuronal axis might be influenced by the so-called *metabolic reprogramming* (MP) [52], *i.e*. the metabolic shift needed to adapt to the demands of an activated state, spelling the transition between different cell specializations or adoption of proliferative/non-proliferative states, either through genetic or epigenetic switches [53]. This phenomenon has been recognized as critically important in lymphocytes, antigen-presenting cells, macrophages, microglia, and astrocytes in different disease scenarios [54, 55]. The most well recognized paradigm of MP is represented by aerobic glycolysis, the *Warburg effect*, first observed in cancer cells [56], but also in activated lymphocytes [57], which shift their main energy metabolism source upon activation to adapt to their differentiated phenotype. Overall, quiescent T-cells, monocyte/macrophages, and B cells are thought to rely on the tricarboxylic acid cycle (TCA) and the electron transport chain for ATP generation. On the other hand, pro-inflammatory activated effector T-cells, which include Th1 and Th17 T-helper populations, as well as activated monocyte/macrophages and dendritic cells, upregulate glycolysis and fatty acid synthesis [58, 59]. Treg effectors are thought to rely to a lesser extent on glycolysis, while committing towards fatty acid and mitochondrial oxidation to fulfill their metabolic needs [58]. It has been suggested that sustained glycolysis might feed pathways such as the pentose phosphate shunt in order to synthesize nucleotides needed for replicating cells, as well as increasing cellular NADPH pools, needed to fuel oxidative burst enzymes, such as NADPH oxidase [60]. It is also worth noting that an MP response is implied in cellular senescence transition, both in cancer microenvironment and in immune cells, where it characterizes cellular exhaustion [61]. Furthermore, MP might also play a role in strengthening inflammatory responses to noxious and non-noxious stimuli through *trained immunity* [62]. MP is thought to pivot on two distinct breaks during the TCA cycle: succinate dehydrogenase (SDH) and isocitrate dehydrogenase (IDH) blockade, leading to succinate and citrate accumulation [63]. The accumulation of metabolites from these metabolic breaks constitutes a signal that translates into transcriptional, epigenetic changes and MP, thus enabling long-lived metabolic effects. The main MP switch is identified in the mammalian target of rapamycin (mTOR) signaling pathway, which in simplistic terms integrates signals such as relative abundance of glucose, amino acids, nucleotides, and fatty acids, to decide cellular commitment towards growth, protein synthesis, and autophagy pathways [64]. The mTOR pathway integrates its activity with NFKB and the hypoxia inducible factors (HIF) pathways in order to tune metabolic activity according to proinflammatory stimuli and oxygen availability [53]. HIF stimulates expression of genes involved in oxidative phosphorylation (OXPHOS) inhibition and glycolysis upregulation by changing the methylation status of hypoxia-responsive elements in their promoter regions [65]. Another reprorammable pathway in activated macrophages, antigen presenting cells, and lymphocytes, is represented by cholesterol metabolism, including its synthesis, uptake, trafficking, and export, which is particularly relevant for phagocytes or cells undergoing clonal expansion [66, 67]. Reprogramming of lipid and cholesterol metabolism is thought to be under the control of SREBP1/2 and PPAR-gamma/PGC-1A signaling [68, 69]. Another relevant aspect of lipid metabolism regarding MP is represented by protein palmitoylation, a post-translational modification thought to occur during cell stress responses, which has been found to affect SREBP1 signaling and several leukocyte functions [70, 71]. In addition to mTOR/HIF-dependent mechanisms, redox signaling, involving the GSH/thioredoxin, NRF2, and nitric oxide cascades, has been linked to metabolic functions in macrophages upon challenge with proinflammatory stimuli, suggesting an important role in MP [72]. Another pathway that might be involved in prolonging proinflammatory MP is represented by NLRP3-inflammasome, which is thought to be upregulated by aberrant glycolysis [73]. MP ultimately leads to long-lasting effects through epigenetic changes induced by cellular and microenvironment signals, such as cytokines, damage associated membrane patterns (DAMPs), and growth factors [53, 74-76]. Intermediate cellular signals acting as MP effectors are represented by metabolites from the TCA cycle, including α-ketoglutarate (A-KG), citrate, succinate and fumarate, but also polyunsaturated fatty acids whose levels fluctuate in response to various stimuli, comprising mTOR and HIF axis activity, redirecting energy production and nutrient uptake according to local demand [63, 77-79]. Experimental evidence suggests that HIF signaling, in addition to its roles in immune cells, exerts on neurons context-dependent dual effects, affecting mitochondrial genesis, antioxidant defenses, and apoptotic pathways [80, 81]. The MP effector metabolites, given their involvement in common pathways between immune and cancer cells, might be termed as immunometabolites or oncometabolites, constituting a continuous spectrum, rather than separate classes. Their role has mostly been studied in terms of cancer microenvironment homeostasis and cancer-related immunosuppression, although research in recent years has highlighted a potentially critical role in autoimmune diseases [82]. As for MS, there is scarce knowledge indicating how MP could constitute a common ground between intrathecal inflammation, neuroaxonal loss and glial dysfunction. Indirect evidence suggests it could be an interesting framework to identify novel therapeutic targets. Teriflunomide, a drug for RRMS, which acts as an inhibitor of the dihydroorotate dehydrogenase enzyme (DHODH), by blocking pyrimidine synthesis, is thought to reduce both mitochondrial respiration and glycolysis in subpopulations of high-affinity autoreactive T-Lymphocytes, reprogramming their metabolism and blocking their activation. Similar effects have also been described on proinflammatory astrocytes by increasing glycolytic metabolism [83, 84]. Glatiramer acetate treatment has also been shown to increase mitochondrial oxidative metabolism and improve antioxidant status in circulating T cells from RRMS patients [85]. Furthermore, disability in MS has been related to exhaustion of CNS remyelinating potential, which, in analogy to immune cells is dependent on cascades such as mTOR, AMPK, and SIRT1, which are heavily implicated in MP [86, 87]. A synopsis of the putative pathogenetic processes involved in MS during the different phases of its course is depicted in Fig. (**1**).

### 1.4 Role of Energy Metabolism and Diet in MS: An Overview

The contribution of immunometabolism to MS pathology constitutes a relatively novel field of research, which could yield insights into new therapeutics through a different mechanistic perspective on the relationship between energy metabolism and MS pathogenesis. Under this perspective, regulating energy metabolism on a systemic scale, either through pharmacological agents or by restricting the availability of certain energy substrates, might represent a viable approach to redirect the outcomes of proinflammatory MP responses. Therefore, dietary interventions might hypothetically represent a way to reconfigure immunometabolism from different angles. In fact, in recent years, several lines of evidence have highlighted the influence of diet composition and diet-derived compounds on immune function and neurodegenerative processes, involving far-reaching effects on brain energy metabolism and synaptic plasticity [88, 89]. Diet has been tightly associated with the development of autoimmune diseases and has been shown to regulate EAE pathogenesis, either by influencing the gut microbiota, T-cell homeostasis, or microglial responses within demyelinating lesions [90]. As for neurodegenerative models, diets with a high content of calories, sugars, and fats, akin to the Western Diet (WD), applied in animal models to induce experimental diabetes, have been reported to induce cognitive dysfunction, especially concerning mnemonic functions [91]. In addition, preclinical models also suggest a negative effect on mitochondrial function through altered reactive oxygen species (ROS) production and respiratory chain protein expression [92]. On the other hand, other regimens, such as Mediterranean Pattern Diets (MPDs), have been reported to improve mitochondrial genesis, dynamics, and respiratory function in both experimental models and human subjects [93, 94]. Diet is thought to intervene in inflammation and neurodegenerative processes at multiple levels, comprising gut microbiota selection, maturation of immune effector cells, as well as immunomodulating or neuroprotective compounds. In Alzheimer’s Disease (AD) and Parkinson’s Disease (PD) models, amyloid/tau deposition and alpha-synuclein pathology are affected. In relation to MS, since the first epidemiological observation that obesity, overweight, and metabolic syndrome are associated with increased incidence and disease activity, diet has been regarded as a useful approach to ameliorate disease activity through overweight prevention and overall health benefits [95]. In keeping with this, diets with a high content of salt, saturated fats, and sugars, consistent with preclinical evidence, have been associated with increased disease incidence and faster progression [96, 97]. Several dietary interventions have shown potential benefits in MS, including ketogenic diet (KD), intermittent fasting diet (IF), low-fat, paleolithic, dietary approach to stop hypertension (DASH), and MPDs. These regimens have been associated with better disease outcomes, suggesting effectiveness in the pathogenesis of autoimmune conditions in small trials [98]. Under a mechanistic perspective, the KD and IF diets have been linked to fat loss, increased insulin sensitivity, and reduced inflammatory cytokine production, including IL-1β, IL-6, and IL-17. Furthermore, KDs are thought to increase autophagy, reduce oxidative stress, and exert a positive influence on gut microbiota [99]. Small clinical studies have reported a benefit of KDs in MS on several clinical measures. The trial from Brenton *et al*. reported beneficial effects on weight paired with a small reduction in disability and fatigue scores in RRMS patients [100]. In accordance with these findings, KD has also been associated with a decrease in circulating neurofilament light chains [101]. Other studies have reported an improvement in anthropometric measures [102, 103]. Consistent with results achieved with KDs, the IF diet has been shown to induce, in two small trials weight loss and an improvement in MS symptoms, such as anxiety, depression, as well as fatigue and cognitive scores [104-106]. Furthermore, in another small trial, a reduction in circulating Th1 cells has been observed [107]. Despite encouraging preliminary results, evidence about the effectiveness of the IF diet on MS is limited by the small sample size and short duration of currently available studies, as well as concerns about its retention on longer time frames [108]. Intriguingly, low-fat diets, which are characterized by a very different composition from KD and IF diets, have also been shown to reduce, in small-sized trials, perceived physical and cognitive fatigue, in addition to promoting weight loss and a reduction in total and LDL cholesterol [109-111]. In a 12-month trial, no differences between low-fat and control diets were found in relapse rate and MRI lesion burden growth [111]. As for the DASH diet, which might represent a variant of the Mediterranean diet, an improvement in fatigue scores and biochemical parameters, such as glial-derived neurotrophic factor levels, has been reported in MS [112]. Another case-control study has suggested an association between this dietary regimen and reduced odds of developing MS [113]. MPDs have been most consistently associated with improved outcomes in chronic neurologic conditions and have been evaluated more extensively in prospective clinical studies regarding the prevention of the most common neurogenerative disorders (*i.e*. Alzheimer’s Disease – AD and Parkinson’s Disease – PD) and MS. A recent network meta-analysis has suggested that both the paleolithic diet and the MPDs might represent the most effective dietary regimens to improve MS quality of life (QoL), therefore they hold a privileged position in this condition [114]. At present, most of the evidence on the effectiveness of diet in neurologic diseases comes from studies regarding the MPDs, which represent the most well-studied paradigms in dietary prevention of neurodegenerative conditions and MS.

### 1.5 Role of MPDs in Neurodegenerative Conditions and MS

MPDs represent a cluster of dietary regimens, that have been adopted by populations inhabiting areas facing the Mediterranean Sea, represented by countries located in Southwestern Europe. The defining characteristics of this dietary pattern are the adoption of whole grains and legumes as the primary source of carbohydrates, fish and low-fat dairy products as protein sources, together with limited red meat and fat intake, paralleled by high consumption of fruit and leafy green vegetables [115]. As for fat sources, fish, olive oil, and nuts are other preponderant components of this dietary pattern, providing mainly polyunsaturated fatty acids. Another variable characteristic of MPDs is the moderate consumption of red wine during meals. Overall, MPDs are characterized by low fat, calorie, and salt content, paralleled by a relatively high content of iron and fiber. Other functionally relevant components are constituted by antioxidant vitamins such as C and E, as well as other classes of antioxidants, comprising various groups of carotenoids and plant-derived polyphenols [116]. The latter two classes of nutrients are not only thought to act as pure antioxidants, since they have been shown to elicit pleiotropic neuroprotective and antinflammatory effects in cellular and animal models [117]. No clear-cut quantitative requirements for MPDs have been defined in scientific literature; therefore, substantial variability between regimens across different studies has been reported [118]. MPDs have been considered since the first observations suggesting reduced incidence of death from cardiovascular diseases in the geographic areas where it is traditionally adopted, the paradigm of a healthy diet [119]. Longitudinal studies have suggested it could help to prevent several chronic diseases, including hypertension, diabetes/metabolic syndrome, autoimmune diseases and cancer, which are characterized by varying degrees of chronic inflammation [120]. In this regard, MPDs have shown to reduce circulating markers of systemic inflammation, the so-called dietary inflammatory index (DII), which comprises IL-1β, IL-4, IL-6, IL-10, TNF-α, and CRP [121, 122], therefore suggesting a widespread modulation of the immune-inflammatory response, arching through proinflammatory mediators, antigen presentation, immune tolerance and memory cell formation [123-125]. Furthermore, growing evidence suggests diet-induced epigenetic effects, such as DNA methylation, histone acetylation/deacetylation and genomic effects [126, 127]. It has been proposed that MPDs mediate protective epigenomic responses by repressing proinflammatory genes, while also potentially contrasting telomere shortening and DNA damage, despite significant inter-individual differences and inconclusive results [126, 128]. Recent evidence from human studies, recapitulating observations from preclinical studies indicate a protective role against neurodegenerative diseases. Both preclinical and clinical studies suggest that MPDs might produce beneficial effects on different neurodegenerative conditions, including PD, AD and MS. AD preclinical models suggest that MPDs can elicit significant changes in the gut-brain axis, increasing the production of microbial metabolites such as lactate. These metabolites positively affect gut permeability, reduce systemic inflammatory markers, and enhance the expression of neuroprotective receptors within the CNS— such as the lactate GPR81 receptor— and lower Tau levels [129]. In analogy to preclinical studies, clinical trials and meta-analyses have highlighted a beneficial role of MPD adherence in ameliorating cognitive symptoms and delaying disease onset [130-132]. Quality of evidence is mainly limited by the observational nature of most studies included, whereas higher fruit and vegetable consumption has been consistently linked to a low, but potentially relevant decrease in disease incidence. Despite the epidemiological association, so far, no consistent association between MPD adherence and progression of brain imaging degeneration markers, such as hippocampal volume or white matter hyperintensities, has been detected [133]. As for PD, in analogy to AD, animal studies suggest that MPDs might be protective by contrasting gut inflammation/dysbiosis and its indirect consequences on the CNS through microbiota selection [134]. In accordance with preclinical models, data from human studies, mainly observational, suggest that MPD might be associated with reduced incidence [135, 136], as well as reduced non-motor symptoms, including cognitive dysfunction [137, 138]. Regarding MS, available clinical studies and meta-analyses linked MPDs to reduced disease incidence, progression, and an amelioration of symptoms such as fatigue and cognitive dysfunction [139]. Several mechanisms have been proposed to explain the potential impact of MPDs in delaying MS onset. Increased vegetable and fiber intake have been proposed, in analogy to AD, PD and other autoimmune diseases, to influence gut microbioma selecting tolerogenic commensals, such as *Bifidobacterium*, *Lactobacillus* and *Streptococcus* species [140], while increasing local short chain fatty acid (SCFA) production, thus affecting epigenetic modulation of the expression of proinflammatory genes [141]. Indeed, depletion of SCFA-producing bacteria, such as *Butyricimonas*, is considered a specific characteristic of MS dysbiosis [142]. Another beneficial factor is represented by the high iron and C vitamin content, which could normalize circulating transferrin and iron levels, whereas iron deficiency and high transferrin levels have been identified as a potential risk factor for developing MS [143]. In addition, disease modifying therapies have also been related to effects on iron homeostasis, potentially reflecting their clinical effectiveness [144, 145]. Under a speculative hypothesis, both diet and disease modifying therapies, interacting with systemic iron metabolism, could reduce CNS iron accumulation, which is augmented in MS and has been linked to neurotoxicity *via* oxidative stress and ferroptosis [146]. As for other dietary factors, alcohol intake has also been linked to dose-dependent immune system modulation through gut dysbiosis, indicating that low/moderate alcohol consumption might increase anti-inflammatory cytokine production and exert antioxidant effects, thereby affecting MS risk [147]. A recent neuropathologic study has reported no association between any alcohol intake and odds of developing MS [148]. In contrast, other observational studies have reported mixed results, which require further enquiry in future studies [149-151]. Lastly, another potential benefit of MPDs in delaying disease onset derives from their role in contrasting insulin resistance and obesity, which could be critical during childhood and young adulthood in triggering MS onset [125]. As for clinical evidence, several studies, since the observation of a high prevalence of MPD in countries with low MS incidence, suggested a plausible role in delaying disease onset and reducing its activity, ultimately reducing disability accumulation [152-154]. Among observational evidence, a recent case-control study by Alfredsson *et al*. has pointed out decreased risk of developing the disease in people adopting MPD in comparison to WD [155]. Notably, another case-control study conducted in Australia detected a reduced incidence of demyelination relapses in patients adopting an MPD enriched with unprocessed red meat [156]. Cross-sectional clinical studies have reported an association between improved QoL, fatigue amelioration, and lower disability [152, 153]. Two recent meta-analyses, including a network meta-analysis, have concluded that MPDs might be associated, despite low-quality evidence, with improvements in perceived fatigue, overall QoL in MS patients [114] and a decrease in circulating inflammatory markers [139]. To date, however, no clinical studies in MS patients have thoroughly assessed the long-term impact of diet on clinical-radiological disease markers; therefore, currently no specific dietary recommendations have been formulated in treatment guidelines. On the whole, MPDs have shown in clinical studies in MS to ameliorate disease symptoms as well as its course [13], albeit their most effective components, along with their mechanism of action, are under scrutiny. Furthermore, it is not yet clear whether MPDs might exert differential effects according to established genetic risk factors, such as HLA DRB1*15:01 or epigenetic markers, like HLADRB1 locus methylation. MP might represent a useful framework to explain some of the diet-related effects, since some of the active components of diet might directly or indirectly influence the activity of MP Master Regulators. Therefore, given the centrality of carbohydrate and lipid metabolism in MP, it could be speculated that dietary interventions might modify immunometabolite levels and thus impact metabolic responses driving MS pathogenesis. Indeed, a potential influence of diet on MP dynamics could be speculated according to recent evidence: a recent study has linked exposure to a WD regimen to a proinflammatory phenotype in myeloid cells [124], suggesting that different diet patterns might promote proinflammatory or antinflammatory effector functions of immune cells through differential metabolism and polarization induction, thus involving MP. In keeping with these findings, a transcriptomic analysis on high-cardiovascular risk patients has highlighted that exposure to MPD might downregulate HIF activity, suggesting similar effects on circulating immune cells [157]. In addition, diet could also indirectly impact MP responses in cells of the innate and adaptive immunity by reducing circulating advanced glycation end products (AGE). AGE receptor activation in CD4^+^ lymphocytes has been found to drive a complex metabolic response, akin to MP [158, 159]. To sum up, most of the available evidence implying a role of diet-derived compounds on immunometabolism derives from studies involving the MPDs and their bioactive compounds, mainly polyphenols, which could affect MP. Given their central role in MPDs, their effects will be discussed in depth in the following section.

### 1.6 Role of MPD Derived Polyphenols on MS Pathogenesis

Polyphenols represent a class of vegetal-derived compounds that are found in plant foods, fruits, cereals, spices, legumes, olive oil, and beverages such as tea, coffee, cocoa, red wine, and herbs. Their chemical structure allows them to protect plants from DNA damage by absorbing UV radiation or scavenging ROS [117]. Several chemical classes of polyphenols are abundant in MPD foods and are mainly represented by flavonoids, phenolic acids, lignans, curcuminoids, tannins, and estilbenzenes [117]. The most well-known compounds, from a pharmacological perspective, are constituted by naringenin, apigenin, kaempferol, hesperidin, ellagic acid, curcumin, oleuropein, hydroxytyrosol, and resveratrol [160]. *In vitro*, polyphenols have been found to engage several biologic targets, interfering with signaling cascades such as mitogen-activated protein kinase (MAPK), phosphatidylinositol3-kinase (PI3K), protein kinase C (PKC), peroxisome proliferator-activated receptor gamma coactivator-1 alpha (PGC1-α), glycogen synthase kinase 3-beta (GSK3-β) while also being able to affect brain-derived neurotrophic factor (BDNF) signaling, reduce P2X7 receptor signaling, NOD-like receptor protein 3 inflammasome (NLRP3) activity, and reversibly inhibit monoaminoxidases [161, 162]. Among other targets, polyphenols are thought to modulate the activity of transcription factors involved in coordinating inflammatory processes and MP, such as AP1, NFKB, and Nrf2 [163, 164]. At present, most evidence suggesting a role of polyphenols in MP has been extrapolated from cancer models and suggests that they might suppress aerobic glycolysis by engaging targets such as PKM2, HK2, GLUT1, and HIF-1 [165], which could also speculatively concur with their anti-inflammatory role in autoimmune diseases. In addition, polyphenols have been shown to support mitochondrial respiration, either by increasing ATP production, mitochondrial biogenesis, or by mitigating oxidative stress in neurons and immune cells [166], therefore eliciting cytoprotective and anti-inflammatory effects in preclinical models of AD, PD, and MS [167, 168]. In AD models, polyphenols have been found to ameliorate amyloid and tau deposition, as well as oxidative stress and cell death [169, 170]. In PD models, consistently with findings obtained with AD models, polyphenols have been found to ameliorate damage caused by oxidative stress, inflammation, ionic imbalance, and alpha-synuclein neurotoxicity [171]. As for MS, preclinical evidence from *in vitro* models suggests that polyphenols, in general terms, might engage several targets in the immune system, encompassing activation of dendritic cells, antigen presentation, differentiation of T cells, as well as phagocytic functions of macrophages [172]. In particular, flavonoids, proportionally with their antioxidant strength, have been found to reduce myelin phagocytosis by macrophages [173]. In addition to their direct effects on redox status and intracellular signaling cascades, polyphenols might influence immune cell differentiation through epigenetic regulation. Epigallocatechin-gallate (EGCG) might inhibit DNA methyltransferase 1, histone deacetylase activity and repress several miRNAs, thus allowing expression of silenced genes [174-176]; curcumin is thought to regulate gene expression through histone acetylase activity [177], while quercetin is thought to favor gene expression through histone acetylase activation, histone deacetylase inhibition [178]. Polyphenols might also exert an indirect positive effect on MS immunopathogenesis by selection of gut microbiota commensals through modulation of the aryl-hydrocarbon receptor/kynurenine pathway, which has been implicated in neurodegeneration, demyelination, and reprogramming towards the antinflammatory Tr1 phenotype in lymphocytes [179-181]. Regarding the effects of polyphenols on MP, Zou *et al*. have found that resveratrol, an important component of MPDs and a putative senolytic, might reduce the activation of pathogenic CD4^+^ T cells [182]. Furthermore, resveratrol has also been shown to inhibit glycolysis in HUVECs and activated CD4^+^ cells, influencing MP [183, 184]. Resveratrol has also been found to upregulate Nrf2 activation in oligodendrocytes challenged with LPS, while reducing NFKB and HIF1A activation [185]. Resveratrol, supposedly through its regulatory action on sirtuins, a ubiquitous class of deacetylases involved in the regulation of cellular metabolism, apoptosis/senescence and autophagy, has been shown to modify several immune cell functions on a metabolic level [186]. Sirtuin activation, either by pharmacological means or by caloric restriction, might stimulate the AMPK signaling cascade while downregulating mTOR activity [187, 188], ultimately promoting autophagy and clearance of dysfunctional mitochondria. Other polyphenols, such as quercetin, luteolin, and EGCG, have also been consistently associated with neuroprotective and anti-inflammatory effects [189, 190]. Similarly to resveratrol, curcumin has been found *in vitro* to regulate T-cell MP, favoring differentiation towards the Treg phenotype through Nrf2 signaling [191]. Hydroxytyrosol, derived from *in vivo* metabolism of oleuropein, has been shown to affect AMPK signaling and autophagy, thus potentially intervening in MP-related signaling [192]. As for preclinical evidence from animal studies, in EAE models, several different polyphenolic compounds have been shown to ameliorate disease severity. Olive oil extract has been found to exert a neuroprotective effect in EAE by engaging SIRT1, increasing expression of antioxidant enzymes,and diminishing proinflammatory M1 microglial activation [193]. Resveratrol has been found to decrease EAE severity, in some models by reducing expression of proinflammatory transcripts [194], in others by eliciting more prominent neuroprotective effects [195]. Resveratrol has also been found to reduce cuprizone-induced demyelination through the improvement of mitochondrial function, oxidative stress, and inhibition of NFKB signaling [196]. Naringenin has also been found to ameliorate EAE by reducing dendritic cell migration and activation of pathogenic Th1/Th17 clones [197]. Similarly to naringenin, apigenin has been shown in different EAE models to reduce adhesion molecule expression and contrast effector cell migration from the periphery to the CNS [198]. Apigenin has also been shown to reduce proapoptotic marker expression in an ethidium bromide-induced demyelination model [199]. On the other hand, ellagic acid has been shown to reduce EAE severity by diminishing inflammation through sphingolipid synthesis, suggesting a neuroprotective potential [200, 201]. Hesperidin injection has also been shown to ameliorate tissue damage in EAE by reducing the expression of proinflammatory cytokines such as IL1β and TNFα and reducing the expression of proapoptotic markers [202]. Kaempferol has been recently shown to improve recovery in a mouse cuprizone-induced demyelination model by reducing proinflammatory microglial activation through reduction of STAT3/NFkB pathway activation and NLRP3 signaling [203]. In analogy with results obtained with other polyphenols, curcumin has shown both neuroprotective and antinflammatory properties in EAE models. Sadek *et al*. have shown that curcumin administration might ameliorate EAE by regulating AMPK/SIRT signaling, leading to increased Nrf2 activity and reduced JAK2/STAT3 pathway activity [204]. Despite their well-established positive effects in preclinical models, evidence from clinical studies is still lacking, with inconsistent results, even though their unfavorable pharmacokinetic profile (low intestinal absorption, rapid excretion) might not allow effective concentrations within the CNS [205]. A exploratory study in MS patients has shown that after green tea administration, polyphenols do not reach detectable CSF levels [206]. Another CSF study has shown that homovanillic acid, 3-hydroxyphenyl-acetic, and caffeic acid could reach detectable levels [207]. As for clinical trials, two recent small-sized trials have assessed the short-term metabolic impact of EGCG on MS patients, suggesting an improvement of muscle carbohydrate metabolism [102, 208]. As for curcumin, a small sized study has shown that a 6-month treatment cycle could decrease circulating Th17 and IL17 cells [209]. A randomized trial assessed the effects of curcumin as an add-on to IFNβ therapy in RRMS patients and, despite a high drop-out rate, suggested efficacy in improving radiological activity markers at 12 but not at 24 months [210]. Another small-sized trial has suggested a positive effect of ellagic acid on depressive symptoms and an improvement of circulating BDNF levels, in MS patients [211]. Overall, considering their pharmacological targets and available evidence, it could be speculated that MPD-derived polyphenols influence inflammatory responses within the CNS through signaling axes, which cross-link with MP, thus sustaining adaptation of immune, glial cells, and neurons to conditions of increased oxidative stress, as occurs during chronic inflammation. It could also be speculated that, given their general effects on mitochondrial respiration and glycolytic enzymes such as HK2 and PKM2, they might influence intracellular levels of immunometabolites relevant to TCA cycle breaks and MP, such as lactate. A synopsis of the putative interactions between diet, immunometabolites, metabolic reprogramming and MS pathogenesis is reported in Fig. (**2**). Considering the hypothesis of diet-driven changes in immunometabolite production, together with current experimental data on MP regarding cancer and immune cells, several molecules appear worthy of a closer assessment of their link to MS pathogenesis, including citrate (CA), itaconate (ITA), ceramides, 2-hydroxyglutarate (2HG), A-KG, glutamate, glutamine, fumarate (FA), SCFA and lactate.

In the following sections, each immunometabolite will be considered in detail regarding its role in MS pathogenesis, as well as available data about the potential relationship with diet. A synthesis of reported studies for each immunometabolite is also reported in Table **1**.

## 2 SEARCH METHODS

A narrative review of the literature involving MPDs, immunometabolism, and Multiple Sclerosis was carried out. A non-systematic Literature search was run on Pubmed and Scopus databases by entering the terms “diet”, “Mediterranean diet”, “Multiple Sclerosis”, “EAE”, “Metabolic Reprogramming”, “Warburg Effect”, “immunometabolism”, “trained immunity”, “autoimmunity”, “neuroaxonal loss”, “neuroinflammation”, “demyelination”, “remyelination”. As for immunometabolites, the terms “fumarate”, “lactate”, “alfa-ketoglutarate”, “glutamate”, “glutamine”, “2 hydroxyglutarate”, “citrate”, “ceramides”, “itaconate”, “short chain fatty acids”, were used. Search terms were combined with Boolean operators to narrow the search field. The reference lists of articles identified by this search strategy were also reviewed, and the working group selected relevant references. In order to obtain a comprehensive overview of this topic, no publication year or article type restriction was applied. A further backward citation search from narrative and systematic reviews identified during the first search was carried out by G.P., C.C., and A.V.

## 3 DIFFERENTIAL ROLES OF MAIN IMMUNOMETABOLITES IN MS PATHOGENESIS

### 3.1 2-Hydroxyglutarate

2 hydroxyglutarate stereoisomers, L-2 hydroxyglutarate (L2HG) and R-2 hydroxyglutarate (D2HG), are produced by reduction of A-KG catalyzed by phosphoglycerate dehydrogenase (PGDH) or by neomorphic isocitrate dehydrogenase 1/2 (IDH1/2) [212]. The latter pathway is thought to be exclusive of cancer cells expressing a mutation in IDH, such as gliomas or haematological malignancies [213]. Conversion of 2HG to A-KG is catalyzed respectively by L-2HG-dehydrogenase and R-2HG-dehydrogenase [79]. Opposite roles have been reported for the two stereoisomers, whereas D2HG has been associated with suppression of immune responses, while L2HG has been associated with proinflammatory properties [55]. D2HG accumulation is thought to reduce inflammation in the tumor microenvironment through metabolic and epigenetic suppression of the immune response, but also through inhibition of enzymes (A-KG dependent dioxygenases, prolyl hydroxylases, histone demethylases, the ten-eleven translocation family of 5-methylcytosine hydroxylases) which influence in cancer cells DNA repair, epigenetic control, response to hypoxia and chemosensibility [214]. In the cancer microenvironment, D2HG has been shown to suppress the activity of cytotoxic T cells by influencing TCR signaling, nuclear factor of activated T cells (NFAT) activity, and polyamine synthesis [215]. On the other hand, D2HG has also been reported to affect Foxp3 gene expression, skewing T lymphocyte differentiation towards a T helper 17 (Th17) phenotype, reducing Treg cells, potentially favoring autoimmunity and chronic inflammation [76, 82, 216]. Blockade of 2HG production has been shown to ameliorate EAE by favoring Treg differentiation [76]. L2HG is involved in downstream TCR signaling in CD8^+^ cells, potentially inducing a memory-like state; furthermore, its production is driven by hypoxic conditions and depends on the HIF axis [217].

As for innate immunity, L2HG has been found to induce proinflammatory signaling and glycolytic metabolism in macrophages, *via* induction of the HIF axis [218]. In addition to its effects on macrophages and lymphocyte differentiation, 2HG has been shown to inhibit microglial activation through the AMPK/mTOR/NF-κB signaling pathway after challenges with proinflammatory molecules, such as lipopolysaccharide (LPS) [219]. Furthermore, L2HG accumulation might result in direct neurotoxicity, since genetic deficits regarding the enzyme L-2 hydroxyglutarate dehydrogenase are responsible for L-hydroxyglutarate aciduria, an autosomal recessive disorder characterized by pediatric onset, widespread myelin damage [220]. Overall, considering its known functions, 2HG appears potentially implicated in various aspects of MS pathogenesis, and its metabolism might constitute a therapeutic target. Evidence from both animal and human studies suggests that dietary approaches might be effective in regulating 2HG production. Animal studies have shown that obese mice display an increased urinary excretion of 2HG [221], while acute myeloid leukemia xenografts harbouring IDH1 mutations implanted in mice fed with a lipid-free diet display reduced 2HG synthesis [222]. In line with this, a recent trial in human subjects has observed that over 1 year, MPD enriched with extra virgin olive oil, in comparison to walnut-enriched MPD and control diet, has been able to reduce plasma 2HG concentration [223]. As for MS patients, studies assessing 2HG levels and their relationship with disease outcomes are lacking, although accumulation of pyruvate and A-KG during fasting has been observed in MS patients, suggesting, under a speculative perspective, that circulating 2HG might be increased [224].

### 3.2 Citric Acid

CA constitutes the main component of the TCA cycle and represents a metabolic *trait-d’union* between oxidation of carbohydrates, amino acids, and fatty acids [225]. Cytosolic CA is exported through the citrate carrier (CIC) and *via* upregulation of ATP-citrate-lyase (ACL) activity, stimulates acetyl-Coenzyme A synthesis, leading to increased fatty acid biosynthesis and protein acetylation, shifting cellular metabolism towards anabolic responses [226]. CA might, either directly or indirectly, inhibit several enzymatic targets, including phosphofructokinase (PFK) 1 and 2, pyruvate kinase (PK), pyruvate dehydrogenase (PDH), and SDH, and is therefore crucial for MP [63, 227, 228]. Mitochondrial CA accumulation, by either IDH or SDH inhibition, might lead to increased ITA synthesis [229, 230]. As for innate immunity, activation of macrophages and dendritic cells by proinflammatory stimuli has been shown to upregulate CIC synthesis, leading to cytoplasmatic CA accumulation, with consequent inhibition of OXPHOS and upregulation of glycolysis and fatty acid synthesis [228, 229, 231]. As for acquired immunity, CA production *via* the TCA has been shown to influence Th17 proliferation, differentiation, and effector functions *via* regulation of glucose metabolism and histone acetylation [77]. EAE studies have reported the occurrence of decreased citrate synthase activity in damaged areas, within the context of mitochondrial complex protein dysfunction [46]. Other EAE models have highlighted that pyruvate dehydrogenase deficient mice, which display a critically reduced CA synthesis, develop a milder disease [77]. In the study from Lee *et al*, ACL expressing astrocytes have been reported to display a persistent proinflammatory phenotype, driven by a long-standing epigenetic memory, leading to increased EAE severity; ACL expressing astrocytes have also been found in MS lesions [54]. In zebrafish adrenal insufficiency experimental models, glucocorticoid therapy has been shown to increase expression of TCA cycle enzymes, including acetyl-Coenzyme A synthase, suggesting a potential mechanistic relationship between TCA activity, CA production, and glucocorticoid anti-inflammatory effects [232]. Zahoor *et al*. have observed that mice treated with the glycolytic inhibitor 2-deoxy-D-glucose displayed a milder EAE phenotype, consistently with the measurement in peripheral blood mononuclear cells from RRMS patients of upregulated glycolysis and significant alterations in the pyruvate and citric acid cycle pathway intermediates with respect to controls [233]. Interestingly, a study from Sylvestre *et al*., has shown decreased CA plasma levels in untreated MS patients with respect to patients on interferon therapy and healthy controls, suggesting a potential relationship with disease activity [234]. Systemic circulating CA is thought to mainly reflect tissue uptake/export, intracellular production, and bone resorption rather than dietary intake [235]. However, its circulating levels might be affected by diet, since a recent study has detected a significant association between MD adherence and higher circulating plasma CA, suggesting a more complex relationship [236]. At present, longitudinal data linking increases or decreases in circulating CA with MS disease activity are not available, although considering preclinical evidence and preliminary biomarker data, CA appears worthy of further enquiry.

### 3.3 Lactate

Lactate, a hydroxycarboxylic acid which can occur in two stereoisomers (L-lactate and D-lactate), constitutes the end-product of glycolysis and is generated by reduction of pyruvate by L or D lactate dehydrogenase (LDH), which also stereospecifically catalyzes conversion of lactate into pyruvate [237, 238]. L-lactate mainly derives from the catabolism of intracellular glucose and alanine, while D-lactate is primarily produced from the transformation of carbohydrates and lipids into methylglyoxal. Further D-lactate metabolism is extensively reviewed elsewhere [239]. It is increasingly recognized that its production does not necessarily reflect anaerobic conditions, since either cancer cells or activated immune cells might upregulate its production in aerobiosis. Innate immunity cells, like macrophages or dendritic cells, as well as lymphocytes, transition from OXPHOS to aerobic glycolysis after being challenged by proinflammatory stimuli. The pleiotropic signaling roles of lactate on cell metabolism, either as an intracellular or circulating messenger, are being increasingly recognized [240]. Lactate has been found to stabilize HIF in tumour-related macrophages [241]. D and L-lactate are not only produced by cellular metabolism, but are also produced by gut lactic acid bacteria, affecting gut permeability and exerting a local immunomodulatory role [242]. Lactate might possess different properties in autoimmune conditions with respect to tumoral models, eliciting context-dependent effects [243]. Cellular models have highlighted that microglia rewire their metabolism towards aerobic glycolysis, thus increasing lactate production, upon challenge with proinflammatory stimuli, such as amyloid β and LPS [244, 245]. As for adaptive immunity, lactate has been found to exert a dual role, acting as an immune-suppressive or as a pro-inflammatory mediator in different cell types. Lactate concentrations have been found to be increased during chronic inflammation, with complex regulatory roles on T and B cells [243]. Pucino *et al*. have observed an important role for lactate in Th17 polarization, which requires glycolysis upregulation [246]. Increased lactate levels appear to prolong tissue infiltration by CD4^+^ and CD8^+^ cells, maintaining chronic inflammation [247]. On the other hand, lactate appears to be required for Treg differentiation, thus exerting an immunosuppressive role [248]. As for B cell immunity, increased lactate production has been linked to the adoption of a senescent secretory phenotype, characterized by increased glycolysis and supportive of chronic inflammation [249]. In animal models, Sanmarco *et al*. have observed that L-lactate ameliorates EAE and dampens the activity of pathogenic dendritic cells in a model of CNS autoimmunity by boosting the HIF-NDUFA42L (NADH dehydrogenase (ubiquinone)-1α subcomplex 4-like 2) signaling axis, thus decreasing mtROS overproduction by complex I [250]. Kaushik *et al*. have observed increased macrophage brain infiltration induced by lactate secretion, as well as heightened LDH expression by resident macrophages from EAE and MS samples, suggesting that enhanced glycolysis might favor CNS demyelination [251]. As for human studies, evidence is mainly limited to biomarker cross-sectional studies. Cerebrospinal fluid (CSF) studies have detected in MS patients an association between increased lactate and disease activity, as well as lesion burden. Serum lactate has also been found to increase in MS patients with respect to controls and in progressive MS with respect to relapsing forms [252, 253]. Lactate CSF concentration might also have a connection with different symptoms of the disease, since its levels have also been associated with the severity of mood disorders [254]. In MS patients, resting plasma lactate levels are increased, while decreased maximal plasma lactate levels after exercise have been observed with respect to healthy controls, especially in patients with progressive disease [255, 256]. Lactate threshold and high intensity interval training regimens have been shown to contrast this alteration, suggesting a potential therapeutic utility [255, 257]. At present, no longitudinal studies have assessed the relationship between diet and lactate levels in MS. Available evidence suggests that lactate might have a direct relationship with MS activity and, therefore, constitute a potential marker in studies assessing the relationship between diet and disease progression.

### 3.4 Itaconate

Itaconate (ITA) is a tricarboxylic acid produced by decarboxylation of TCA cycle intermediate cis-aconitate by cis-aconitate decarboxylase (ACOD1), which is encoded by the IRG1 gene, whose expression is restricted to myeloid lineage cells [258]. ITA synthesis is upregulated during inflammatory responses against pathogens, whereas it shows antimicrobial, antiviral, and anti-inflammatory properties [74]. Its effects on inflammatory signaling take place at several levels, either as an intracellular signal or as a secreted signal. On the whole, ITA is considered a potent mediator of MP, with a preponderant anti-inflammatory role [259]. Firstly, competitively inhibiting SDH induces succinate accumulation, tuning mitochondrial respiration and ROS production in macrophages [228]. It also stimulates cellular stress response cascades by activating nuclear factor erythroid 2-related factor 2 (NRF2), activating transcription factor 3 (ATF3), and inhibiting NLRP3 [260]. ITA is considered a glycolytic inhibitor by alkylating cysteine residues of several metabolic enzymes, including glyceraldehyde 3-phosphate dehydrogenase (GAPDH) [260]. It has been heavily implied in MP in lymphocytes, reducing polarization towards the Th17 cell type while increasing Treg numbers, through downregulation of glycolysis and OXPHOS mediated by genetic and epigenetic modulation. In addition, ITA ameliorates EAE severity [74]. Furthermore, ITA has also been found to influence macrophage effector functions and polarization, suggesting cell-specific and context-dependent effects [228, 261]. Other relevant downstream targets are constituted by the STING/NFKB axis and the HIF 1A (HIF 1A) cascade, influencing the type I interferon response [228, 262]. ITA might also affect innate immunity functions by interaction with its transmembrane G-protein coupled receptor, OXGR1, which also responds to AKG and has been implicated in responses against bacterial pathogens [258]. Recently, ITA accumulation in macrophages has also been implicated in explaining anti-inflammatory properties of glucocorticoids [263]. Its emerging role in autoimmune conditions is supported by evidence linking its reduced production to excessive inflammation, such as in rheumatoid arthritis [264, 265]. As for MS, growing evidence from animal models suggests a potential role in shaping disease severity. Recent studies on mouse models have highlighted that boosting ITA levels ameliorates EAE severity, recapitulating results obtained on macrophages, by dampening uncontrolled inflammatory responses from microglial cells and reducing expression of proinflammatory markers, such as CD80 [266, 267]. In addition, multilineage cell culture models involving macrophages, mixed glial and hippocampal neurons have shown that dimethylfumarate, a drug approved for RRMS, contrasts, similarly to ITA, NLRP3 activation, limiting IL-1 secretion [268]. ITA and dimethylfumarate have also been shown to contrast, in human microglial cell models, MP induced by proinflammatory stimuli, by reducing glycolysis and increasing mitochondrial metabolism [269]. As for whole-body metabolism, data from mouse models, despite conflicting evidence, suggest that ITA might induce fat oxidation, thermogenesis, and increase glucose tolerance [270]. Dimethyl-itaconate, a cell-permeable ITA analogue, has been shown to ameliorate in mice cognitive dysfunction induced by a high-fat diet (HFD), by counteracting diet-induced gut dysbiosis [271], while Eberhart *et al*. in their study have linked increased ITA production to HFD-induced gut dysbiosis [272], suggesting further enquiry. At present, no studies have directly assessed the relationship between dietary regimens and ITA in human subjects. In a sub-analysis of the PREDIMED study, plasma cis-aconitate, the ITA precursor, has been shown to increase after one year of adherence to olive oil or nut-enriched MPD [273], suggesting that ITA levels might be indirectly affected. Therefore, considering the current preclinical evidence and the well-established epidemiological relationship between obesity, metabolic syndrome, HFD, and MS, there could be a rationale for evaluating ITA dynamics in response to diet and immunomodulatory therapies in MS.

### 3.5 Short Chain Fatty Acids

SCFAs, mainly represented by propionic, butyric, and acetic acid, have been recently recognized among the most relevant microbial metabolites produced by the gut flora with the ability to tune local and systemic immune responses [274]. Other SCFAs, such as pentanoate and valerate, are also being increasingly recognized as potent regulators of immunometabolism [275]. SCFAs are mostly produced by anaerobic gut commensals by fermentation of complex carbohydrates, especially dietary fiber, while in minor proportions derived from protein catabolism and other dietary sources [275, 276]. SCFAs might activate, with an affinity ranging from high micromolar to low millimolar concentrations, G-Protein coupled receptors such as FFAR2 and FFAR3, which are highly expressed in innate immune cells, but not in lymphocytes, and are involved in host defense. [277, 278]. A part of their immunomodulatory effects have been linked to their ability to modulate Th17 responses as well as induce Treg differentiation through an epigenetic switch requiring histone deacetylase (HDAC) inhibition. [279]. Polyunsaturated and SCFAs might activate G-protein coupled receptors (GPR) like GPR35, GPR43, GPR120, which are involved in HDAC inhibition and transcriptional activation [13, 279, 280].

Acetate has been linked to suppression of anti-tumoral CD8 responses through metabolic reprogramming in lymphocytes, while butyrate has been associated with increased cytotoxic responses [281, 282]. Other potentially relevant mechanisms are constituted by reinforcement of epithelial intestinal permeability, thus preventing bloodstream transfer of cross-reactive antigens or autoreactive cells [283]. Evidence from MS animal models suggests a potential protective role. Loss of intestinal mucosal integrity with increased permeability has been observed as a precocious feature during EAE induction, constituting therefore a potential therapeutic target [284]. Propionate supplementation during EAE induction elicited tolerogenic lymphocyte polarization in the gut and spleen, ameliorating disease course [285]. Furthermore, SCFAs, in addition to their *in loco* effects, have also been reported to dampen inflammatory microglial responses through NFKB regulation and epigenetic tuning [286]. Evidence from human studies appears to support the hypothesis of a protective role of SCFAs. In MS patients, lower levels of circulating serum/plasma propionate, acetate, and butyrate have been measured, correlating with follicular helper T cell subsets [287, 288]. Furthermore, in a small pilot study from Haase *et al*., fecal propionate levels were found to be reduced in MS obese patients, while circulating Th17 cells were increased. In the same patients, 3-month propionate supplementation was able to increase the Treg/Th17 ratio [285]. Another proof-of-concept study detected an increase in Treg numbers and suppressive capacity beginning after two weeks of propionate supplementation, paralleled by an amelioration in clinical relapse, progression rate, and radiological markers of CNS atrophy in MS patients after a three-year follow-up [142]. MPD, as well as other fiber-enriched diets, might increase circulating SCFAs, contrasting sustained chronic inflammation. Increasing SCFA signaling might therefore constitute a simple and effective strategy to target chronic inflammation in MS. Determination of their changes over time might also be considered a biomarker for future studies assessing the effects of diet on disease progression.

### 3.6 α-Ketoglutarate/Glutamate/Glutamine Axis

Glutamate, while being mainly acknowledged as the most important excitatory neurotransmitter of the CNS, has, in recent years, been “rediscovered” as an immunometabolite, acting in synergy with AKG and glutamine. Glutamate has been theorized to exert complex regulatory roles on inflammation and immunity, either by activation of its ionotropic and metabotropic receptors, or by modulation of cell energy metabolism through anaplerotic reactions [289]. Glutamate might enter cells through ubiquitous transmembrane transporters, or may be synthesized from glutamine by glutaminolysis or conversion from AKG, catalyzed by glutamate dehydrogenase (GDH) and aspartate aminotransferase (AAT) [290]. Glutaminolysis is considered a crucial pathway for energy production in lymphocytes [291], which reconfigure their metabolism towards glycolysis and glutaminolysis upon antigenic stimulation through a Myc-dependent gene expression switch [292]. Glutaminolysis is required for T cellgrowth upon activation and has also been linked, through production of AKG, to increased macrophage polarization towards an M2 phenotype [79, 292]. As for CD4 lymphocytes, experimental studies have shown that increasing AKG levels might alter DNA and histone methylation states [293] and, through conversion into 2HG, might increase the methylation of the *Foxp3* gene locus, thus influencing Treg polarization [76]. On the other hand, increased AKG might stimulate OXPHOS and, through an SDH-dependent switch, lipogenetic activity, sustaining CD4^+^ helper effector functions [293]. AKG might also be decarboxylated by reacting non-enzymatically with hydrogen peroxide, dampening oxidative stress and producing succinate [294]. AKG supplementation might affect intestinal innate immunity through intestinal microbiota composition and inhibiting intestinal villi apoptosis [295], hence maintaining gut integrity [296]. Therefore, AKG dietary supplementation has been regarded as a potential strategy to treat age-related diseases, although data from clinical studies at present are lacking [297]. Glutamate, despite its potential role at supraphysiological concentrations as an excitotoxic signal, might also fuel energetic metabolism through anaplerotic reactions during hypoxic conditions, probably involving AKG increase [298]. Glutamate and glutamine metabolism undergo a significant rewiring during lymphocyte activation, leading to decreased glutamine oxidation and increased intracellular concentrations of alanine, pyruvate, and lactate, suggesting the occurrence of a metabolic shunt [299]. In EAE, several alterations in glutamate degrading enzymes, glutamate transporters, glutamate receptors, and glutamate signalling have been reported in the CNS [300-302]. As for immunologic aspects, lymphocytes from MS patients display an abnormal activation in response to glutamate, partly mediated by increased GluR3 expression, which is increased in patients with either radiologically or clinically active disease [303]. Furthermore, glutamate might promote inflammation by augmenting T-cell activation and chemotaxis through AMPA (gluR3) receptor signalling, but also by increasing lymphocyte proliferation [33]. Notably, glutamate might also exert a counterbalancing action mediated by selective mGLUR4 activation, which has been linked to EAE amelioration through increased Treg responses [34, 304]. As for glutamine, evidence coming from EAE experimental models is conflicting . The study from Palmieri *et al*. has reported an association between reduced tissue expression of the enzyme glutamine synthase, with a consequent relative decrease in the glutamine/glutamate ratio and increase in disease severity correlating with microglial inflammatory activity [305]. The study from Hollinger and colleagues, on the other hand, has shown that administration of the glutaminase inhibitor (JHU-083) suppresses T cell activation, proliferation, and attenuates EAE severity, with no significant effects on dendritic cell activation [306]. In line with these findings, Kono *et al*. have reported reduced EAE severity in mice treated with a glutaminase-1 inhibitor mediated by reduced T cell glycolysis, HIF1A activation, and Th-17 differentiation [307]. At present, it is not well-known whether different dietary approaches might affect systemic glutamate or glutamine homeostasis, which are metabolites with a very fast turnover. As for glutamate, a recent study has shown that short-term implementation of MPD in subjects affected by Mild Cognitive Impairment might modulate, either in neurons or in peripheral mononuclear cells, gene expression of several transcripts involved in glutamatergic signaling, comprising glutamate receptors and transporters, while also improving amyloid/tau pathology markers and circulating neurofilament light chain levels [308]. In MS patients, increased plasma glutamate levels with respect to healthy controls have been measured, while Sarchielli *et al*. in CSF samples have measured higher glutamate levels in MS patients with respect to controls, increasing in clinically active and progressive forms in comparison to stable patients [309, 310]. According to currently available evidence, MPD adoption was not associated with significant variations in circulating glutamate [311], even though the hypothesis of a potential influence on glutamatergic signaling or glutamate-related metabolic reprogramming cannot be ruled out, suggesting the conduction of further studies.

### 3.7 Fumarate

FA, an intermediate of the TCA cycle, is mainly produced by the oxidation of succinate catalysed by SDH. FA might subsequently either be transformed into malate by fumarate hydratase (FH), thus fueling NADH synthesis, or be shunted towards cellular cysteine succinylation, or block 2-OGDD enzymes. The involvement of SDH in both the TCA cycle and the electron transport chain renders FA synthesis a crucial regulatory step of aerobic metabolism, which is involved in MP [229]. The role of FA in MP has been more extensively studied in cancer cells, where rising cytoplasmic FA levels after inhibition of FH might inhibit PHDs and activate the HIF axis [78]. Loss of FH activity is considered to promote survival of cancer cells by allowing FA accumulation: tumour-derived FA is thought to inhibit infiltration of CD8 T-cells while also favouring epithelial to mesenchymal transition, favouring cancer progression [312]. Furthermore, FH deficiency is responsible for a neonatal-onset epileptic encephalopathy characterized by CNS hypomyelination [313]. As for innate immunity, intracellular accumulation of FA, which occurs upon LPS macrophage activation, is thought to require upregulation of the argininosuccinate pathway and has been linked to several functions, such as IL10 secretion, TNF secretion, or type 1 interferon responses [314, 315]. FA, in analogy to ITA, might activate the NRF2 axis by inducing modification of cysteine residues, thus upregulating antioxidant/cytoprotective defenses [316], but also contrasting inflammasome assembly, by inhibiting, through succinylation, gasdermin D oligomerization and IL1β synthesis [317]. Most data from animal models of MS regard FA esters, such as monomethyl fumarate (MMF) and dimethylfumarate (DMF), which are thought to mimic immunological effects of FA accumulation. DMF has been shown to ameliorate EAE severity, either through neuroprotective effects carried out by upregulation of antioxidant defenses or through their immunomodulatory actions. In particular, DMF has been shown to stimulate Treg differentiation in EAE models, albeit after an initial pro-inflammatory response [318]. DMF, which constitutes an approved drug for RRMS, is thought to induce, in analogy to FA, succinylation of intracellular cysteines, inhibiting the enzyme GAPDH and, therefore, inhibiting glycolysis and increasing OXPHOS in myeloid cells, while dampening activation of certain subpopulations of autoreactive lymphocytes [319]. DMF has been found to promote in MS patients an increase in Tregs, CD56^bright^ natural killer cells, plasmacytoid dendritic cells, and a decrease in CD8^+^ T cells, B cells, and type 1 myeloid dendritic cells [316]. Furthermore, FA esters, in MS patients, were found to alter the expression of chemokine receptor CCR6 in CD4^+^ and CD8^+^ T-cells, while also inducing DNA methylation changes in Th17 cells, inactivating the proinflammatory microRNA MIR-21 promoter [75]. On the whole, in analogy to ITA, FA is mainly regarded as an anti-inflammatory/immunosuppressive metabolite in MS. No studies at present have assessed, however, the relationship between circulating FA and disease severity or activity, although mesenchymal stromal cells from MS patients display mitochondrial FH loss, which has been linked to a reduced neuroprotective potential [320]. At present, it is not clear whether diet might significantly affect systemic FA or its metabolism in immune cells. As for MPD, in the PREDIMED study, MPD adoption was not associated with significant changes in circulating FA [223].

### 3.8 Ceramides

Ceramides are long-chain lipid molecules produced in sphingolipid metabolism, which are involved in a wide variety of cell signaling processes, including inflammation and cell death [321]. They are produced through three different pathways, represented by de novo synthesis, catalyzed by the enzyme serine palmitoyl-transferase, direct hydrolysis from sphingomyelins, catalyzed by sphingomyelinases, and the so called salvage pathway, which occurs in lysosomes, where different sphingolipids, including glucosylceramides, and sphingomyelins can be converted to ceramides [322]. Circulating ceramides are thought to preponderantly derive from the *de novo* synthesis pathway, which is highly dependent on dietary intake of serine and/or palmitate and may be upregulated by stress and high circulating oxidized low density lipoproteins [322, 323]. Ceramides are identified according to carbon chain length (which usually does not exceed 24 atoms in mammals), hydroxylation, and presence of double bonds, which confer them distinct biological functions [324, 325]. Sphingosine, especially in its phosphorylated form (sphingosine-1 phosphate) and ceramides (which might also undergo phosphorylation) are thought to exert opposing effects on cell metabolism and survival cascades, mainly through the PI3K-AKT-mTOR and autophagy axes [326], but also through epigenetic mechanisms, comprising histone deacetylation [327]. Their hormetic signaling constitutes, therefore the so-called “sphingolipid rheostat”. The relevance of sphingolipid signals has been recognized in cell death regulation, but also in immunometabolism and autoimmunity. Ceramide production by neutral sphingomyelinases has been suggested to induce MP in T cells, dampening early overactivation of these cells, allowing them to sustain prolonged activation [328]. In particular, increased circulating C16 ceramides have been measured in untreated patients with neurotropic autoimmune diseases such as systemic lupus erythematosus, with higher levels associated with more aggressive disease [329]. In addition to their role in immune processes, their direct neurotoxic properties suggest a pathogenetic role in neurodegenerative diseases. In EAE models, sphingosine and ceramide accumulation have been linked to increased disease severity, partly mediated by increased inflammation, partly by a direct neurotoxic effect of certain sphingolipids [330]. It has been shown that specific ceramides (C:16; C:24) isolated from CSF of MS patients might produce mitochondrial impairment on cultured neurons [331]. The same ceramides have also been associated with increased cardiovascular risk. A recent drug class introduced in MS therapy is constituted by sphingosine-1 phosphate receptor (S1PR) modulators, including fingolimod, siponimod, ponesimod, and ozanimod [332]. In addition to their inhibition of lymphocyte migration towards the CNS, resulting in a lesser degree of compartmentalized inflammation, S1PR modulators have shown, in experimental models, to exert neuroprotective properties by interacting on neurons, astrocytes and oligodendrocytes, reducing mitochondrial dysfunction, oxidative stress, BBB dysfunction, but also upregulating production of neurotrophic factors [333, 334]. Furthermore, fingolimod administration has been found to ameliorate ceramide-induced pro-inflammatory signaling in reactive astrocytes [335]. Siponimod and ponesimod in clinical trials have been shown, respectively, to modify disability accumulation in PMS and brain atrophy progression, suggesting neuroprotective effects [336, 337]. In addition to their intracellular signaling roles and their potential structural function coming from plasma membrane incorporation, ceramides might also affect energy metabolism balance. In fact, ceramides have been associated with increased cardiovascular risk, but also to impairment in glucose metabolism and obesity [338]. Circulating ceramides are thought to be heavily influenced by dietary habits, although evidence at present is conflicting. It has been suggested that low-fat diets might achieve sustained reduction in plasma ceramide levels. In the PREDIMED study, a MPD intervention failed to achieve, at one year, a reduction on circulating ceramides [339], while the FRUVEDomic study reported a reduction in C24 and an increase in C16 after implementation for 8 weeks of a dietary regimen characterized by low-fat and refined carbohydrate content associated with high intake of fruit and vegetables [340]. Factors like intervention duration might impact this association, since the Framingham offspring cohort study reported a reduction in circulating C16 in patients displaying the highest adherence to a MPD [341]. At present, it is not well-known whether circulating increased ceramides might represent a detrimental or a protective response in MS, although the reduction of their concentration to physiological levels might speculatively constitute a biomarker of therapeutic response, worthy of further enquiry in clinical trials assessing either drug or dietary intervention efficacy.

## 4 DISCUSSION

MS can be considered a chronic inflammatory disease of the CNS characterized by overlapping autoimmune demyelination and neurodegenerative processes that ultimately converge on neuroaxonal loss. Cellular energy metabolism appears to play a critical role throughout the entire disease course, highlighting the importance of preserving mitochondrial oxidative metabolism in all involved cell types. Early in the clinical course, despite subtle degenerative changes in normal-appearing gray and white matter, inflammatory demyelination driven by T cell responses is the primary driver of disease. In later phases, however, the combined effects of chronic *smoldering*, inflammation, and insufficient remyelination contribute to synaptic dysfunction and neuroaxonal loss. Mitochondrial energy production impairment and structural damage, amplified by the proinflammatory environment, lead to a condition of *relative hypoxia*, inducing activity of metabolic stress cascades in nerve cells. Activated lymphocytes, macrophages, and microglial cells, upon challenge by proinflammatory stimuli, rewire their metabolism, ultimately reducing OXPHOS and heightening glycolysis as occurs in cancer cells in response to hypoxic conditions. These metabolic changes require MP, which pivots on master regulators of metabolism and growth such as mTOR, AMPK and, most relevantly, the HIF1A axis, a ubiquitous response mechanism to metabolic stress, which increases aerobic glycolysis in immune effector cells and glial elements, but could also represent a shared switch between autoimmunity and cell death cascades of nerve cells. In fact, HIF1A activation might, at the time, stimulate chronic inflammation and increase mitochondrial dysfunction within demyelinating foci within the CNS, rendering nerve cells more susceptible to damage. In configuring HIF1A activation during MP immunometabolites originating from the TCA cycle, such as ITA, may play a critical role. It could therefore be assumed that controlling their levels could diminish MS pathology by acting on multiple targets. Under a speculative perspective, despite evidence coming mostly from preclinical studies, it could be hypothesized that distinct metabolites, despite their overlapping roles and their potential cell-specific functions, may have differential effects on disease pathogenesis. 2HG appears as a proinflammatory signal whose production should be reduced in order to control excessive Th17 cell polarization. Lactate appears mostly as a proinflammatory signal, whose increase might be tied to chronic inflammation but also functional CNS hypoxia, despite being associated with a protective gut microflora. CA, on the other hand, appears as a master regulator of Th17 responses and, therefore, appears as a detrimental proinflammatory signal. ITA and FA signaling axes appear as potent antinflammatory, tolerogenic, and neuroprotective mechanisms when prevailing on succinate signaling. C16 and C24 ceramides, instead, appear as neurotoxic and proinflammatory signals. The Glutamate-Glutamine-AKG axis could also represent a protective anaplerotic mechanism, despite conflicting evidence, if not paralleled by overstimulation of other proinflammatory pathways such as succinate or citrate signaling. Ultimately, SCFAs appear to promote in a pleiotropic manner tolerogenic responses, mainly through microbiota selection. Immunometabolites could also interfere with the response to several disease modifying therapies. β−interferons might act in synergy with anti-inflammatory signals such as ITA and FA in HIF-STING mediated responses. Glatiramer acetate appears to stimulate OXPHOS in circulating CD4^+^ T-cells. DMF, on the other hand, might strengthen ITA and FA signalling, thus stimulating Treg genesis. Teriflunomide appears to reduce the metabolic activation pattern of rapidly proliferating T cells, while S1PR modulators might contrast the detrimental effects of excessive ceramide signalling on the CNS, such as proinflammatory astrocytic responses. Other approved drugs, such as ocrelizumab and natalizumab, do not appear to interact with metabolic patterns of distinctly polarized cell subtypes. However, immune-reconstituting drugs such as alemtuzumab and cladribine, by removing a significant fraction of the immune cell repertoire, might induce the remaining cell clones to redirect their metabolic profile towards biosynthetic rather than effector pathways. On the whole, immunometabolism might hypothetically be influenced by several elements, including environmental factors such as smoking, alcohol consumption, and diet. Certain dietary regimens have indeed been shown to reduce markers of chronic inflammation in MS. Among these, MPDs are the ones who have yielded the most consistent longitudinal results on reducing disease incidence and symptoms. Despite the potentially numerous mechanisms of action of dietary intervention in affecting autoimmunity and neurodegenerative processes, it could be speculated that diet-induced fluctuations in immunometabolite levels and MP tuning might contribute to the beneficial effects observed on MS pathogenesis. Considering MPDs, immunometabolite levels could speculatively be affected by polyphenols, which exert pleiotropic effects on intracellular signaling cascades involved in cell proliferation and MP and have been shown in preclinical studies to support mitochondrial dysfunction and reduce aerobic glycolysis. On the other hand, minimal evidence currently supports that MPDs could affect the production of metabolites involved in MP, especially citrate. At present, no evidence linking increased circulating citrate to MS phenotype and outcomes is available, suggesting further studies. Nonetheless, the hypothesis of a significant impact of diet on MP dynamics could be supported by the observation that MPDs might downregulate systemic HIF1A activation and ceramide production, suggesting a potential role in counteracting their effects in the CNS and immune cells, which could be worthy of further enquiry.

## CONCLUSION

Immunometabolism and MP appear as crucial pathways in MS, as well as a potential layer of diet-related immunomodulatory effects, which could disclose shared pathogenic mechanisms. At present, several questions arise on immunometabolites, especially regarding their roles in CNS cells and the eventual coexistence of common metabolic reprogramming trajectories between innate, adaptive immunity, and the glio-neural axis. Further preclinical studies are needed on this subject, especially regarding toxin-induced models of demyelination, in order to assess whether immunometabolites and MP might play a direct role in the response to neurotoxic insults in nerve cells, in addition to their immunologic effects. Similarly, animal studies assessing how specific diet patterns/components might influence immunometabolite production in MS models, either in circulating or in CNS-resident cells, would be needed in order to acquire mechanistic insights. At present, evidence coming from human studies suggests that the therapeutic effect of diet on MS pathogenesis could involve immune effector cell metabolism and polarization, but also selection of a protective microbiota and a reduction in overall systemic inflammation. Currently available evidence suggests that MPDs could modify circulating immunometabolites while tuning systemic HIF1A axis activation. Despite the preponderant anti-inflammatory action of MPDs, it is still not clear whether these effects could be involved in their beneficial actions against MS development and symptoms. Specific trials addressing the impact of MPD interventions on MS patients, adopting longitudinal measurement of immunometabolites while assessing the relationship with functional characteristics of circulating/intrathecal immune cells, would be needed to increase knowledge about this subject. Whether immunometabolic markers might characterize the relationship between energy metabolism and radiological progression or disease events such as relapses, clinical progression, or absence of disease activity is still under debate, although current preclinical evidence might support this view. Therefore, under this perspective, diet-induced changes in immunometabolism could represent an adjuvant therapeutic measure, which could increase the effectiveness of disease-modifying or immune-reconstituting drugs. In addition, modifying immunometabolism might also, from a speculative perspective, have a beneficial effect on cognitive symptoms, fatigue, or mood disturbances in MS patients by ameliorating the dysfunctional crosstalk among the cells involved in the pathogenetic process, although further studies are needed.

## Figures and Tables

**Fig. (1):**
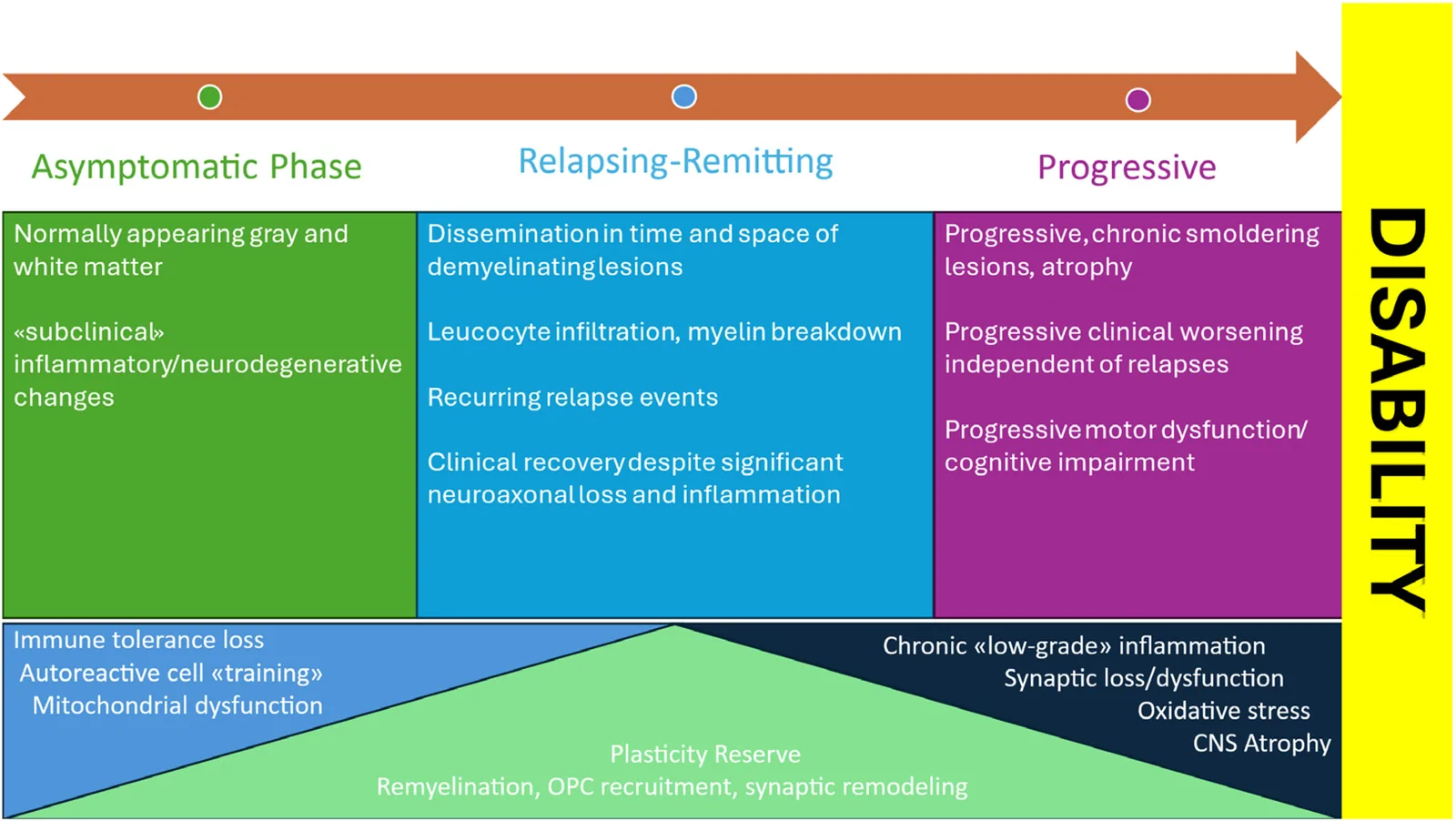
Synopsis of MS natural history, clinical course, and pathogenesis.

**Fig. (2):**
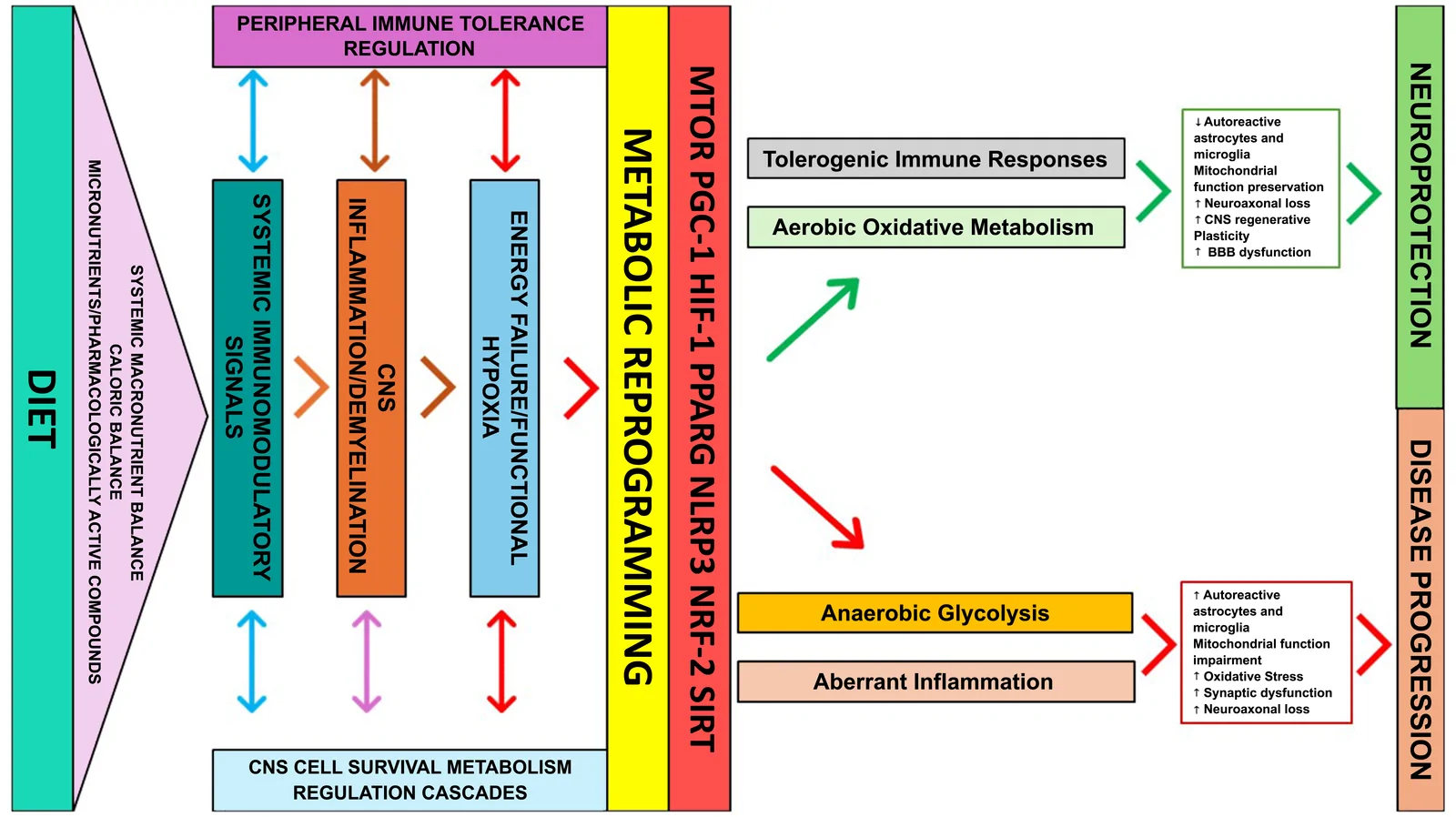
Interactions between diet, immunometabolites, MP and CNS pathology in MS.

**Table 1:** Overview of current evidence from preclinical and clinical studies on immunometabolites.

**2-Hydroxyglutarate (2HG)**			
**Study**	**Study Type**	**Cell Type/Model**	**Effect**
Bunse *et al.* 2018 [215]	Preclinical *in vivo* (mouse)	CD4+, CD8 +T cells	T-cell mediated immunity suppression by D2HG (NFAT, TCR, polyamine signaling)
Xu *et al.* 2017 [76]	Preclinical *in vivo* (mouse)	T cell cultures EAE	Increased cell polarization towards Th17 subtype by D2HG Reduced severity by D2HG reduction
Tyrakis *et al.* 2016 [217]	Preclinical *in vivo* (mouse)	CD8+ T cells	Memory-like state induction
Williams *et al.* 2022 [218]	Preclinical	Macrophages	HIF induction, proinflmmatory signaling, glycolysis upregulation
Han *et al.* 2019 [219]	Preclinical *in vitro* (cell model)	Microglia	AMPK-mediated suppression of LPS-induced proinflammatory signaling
Santos *et al.* 2023 [223]	PREDIMED clinical Trial subanalysis	Plasma biomarkers	Reduced circulating 2HG by Olive-oil enriched MPD
**Citric Acid (CA)**			
Everts *et al.* 2014 [227]	Preclinical *in vivo* (mouse)	Dendritic Cells	Glycolysis induction, MP
Lampropoulou *et al.* 2016 [228]	Preclinical *in vivo* (mouse)	Macrophages	Glycolysis induction, MP
Soriano-Baguet *et al.* 2023 [77]	Preclinical *in vivo* (mouse) – *in vitro*	EAE Murine CD4 Th17 cells	Decreased CA induces EAE amelioration Decreased CA reduces Th17 polarization (reduced glycolysis and glutamine utilization)
H.G. Lee *et al.* 2024 [54]	Preclinical *in vivo* (mouse) *in vitro* (cell model) Post-Mortem Immunihistochemistry	EAE Astrocyte cultures (mice-human) MS autoptic samples	ACLY inactivation leads to EAE amelioration ACLY inactivation abrogates persistence of a proinflammatory phenotype *via* an epigenetic mechanism ACLY expressing astrocytes are highly present in MS lesions
Weger *et al.* 2016 [232]	Preclinical *in vivo* (zebrafish)	Zebrafish adrenal insufficiency model	Glucocorticoid-induced upregulation of TCA enzymes
Zahoor *et al.* 2022 [233]	Preclinical *in vivo* (mouse) Human Metabolomic	EAE RRMS PBMC	Glycolysis inhibition induced EAE amelioration Altered TCA, pyruvate cycle; upregulated glycolysis
Sylvestre *et al.* 2020 [234]	Human Metabolomic (cross-sectional)	RRMS Plasma Metabolomics	Decreased CA levels
Macias *et al.* 2019 [236]	MEDDINI Clinical Study	Plasma Metabolomics	Increased CA levels in patients adherent to MPD
**Lactate**			
Sanmarco *et al.* 2023 [250]	Preclinical *in vivo* (mouse) – *in vitro*	Dendritic Cells EAE	L-Lactate decreases mtROS production by stimulating the HIF1-NDUFA42L axis L-Lactate and D-lactate administration ameliorate EAE
Kaushik *et al.* 2019 [251]	Preclinical *in vivo* (mouse) – *in vitro* Post-Mortem Immunohistochemistry	EAE Macrophage cultures MS Samples	Blockade of Lactate secretion by macrophage infiltration ameliorates EAE pathology Blockade of lactate production/secretion dampens macrophage chemotaxis Increased LDH expression in perivascular infiltrating macrophages
Voloboueva *et al.* 2013 [245]	Preclinical *in vitro*	BV-2 Microglia	Increased lactate production in BV2 cells challenged with LPS
Rubio-Araiz *et al.* 2018 [244]	Preclinical *in vitro*	Neonatal Mice microglia	Increased lactate production in microglial cells stimulated with LPS + Abeta
Pucino *et al.* 2019 [246]	Preclinical *ex vivo*	Human T lymphocytes	Lactate promotes glycolysis and persistent inflammation
Haas *et al.* 2015 [247]	Preclinical *in vitro*	CD4^+^ CD8^+^ T Lymphocytes	Lactate promotes T cell entrapment in peripheral tissues
Angelin *et al.* 2017 [248]	Preclinical *in vivo* (mouse) – *in vitro*	Mice T lymphocytes	Lactate increases Treg polarization
Romero *et al.* 2024 [249]	Preclinical – *ex vivo*	Human B cells	Lactate promotes a secretory immunosenescent phenotype
Lutz *et al.* 2007 [252]	Human Metabolomic (cross-sectional)	CSF from RRMS patients and HC	Increased lactate levels in active disease
Esmael *et al.* 2021 [253]	Human biomarker (cross-sectional)	Plasma from HC vs RRMS vs PMS	Increased
Amato *et al.* 2021 [255]	Single-arm non randomized clinical trial	MS patients	Reduced plasma lactate after training
Cerexhe *et al.* 2022 [256]	Systematic Review and Meta-analysis	Human biomarker studies	Increased resting state plasma lactate in MS with respect to controls. Decreased Lactate production during exercise.
Zaenker *et al.* 2018 [257]	Single-arm non randomized clinical trial	MS patients	Decreased lactate production after exercise
**Itaconate (ITA)**			
Aso *et al.* 2023 [74]	Preclinical *in vivo* (mouse) – *in vitro*	Murine T Lymphocytes EAE	Itaconate diminishes Th17 polarization and increases Treg polarization Itaconate ameliorates disease severity
Runtsch *et al.* 2022 [259]	Preclinical *in vivo* (mouse) – *in vitro*	Macrophages	Decreased polarization towards proinflammatory phenotypes
Lamproupolou *et al.* 2016 [228]	Preclinical *in vivo* (mouse)	Macrophages	SDH inhibition, MP induction, anti-inflammatory
Mills *et al.* 2018 [262]	Preclinical *in vitro* – *in vivo*	Human macrophages	Nrf2 activation, type I interferon response modulation
Auger *et al.* 2024 [263]	Preclinical *ex vivo* – *in vitro*	Human macrophages/murine macrophages	Glucocorticoids contrast MP through ITA dependent signaling
Kuo *et al.* 2020 [266]	Preclinical *in vivo* (mouse)	EAE	Attenuated severity
Zhao *et al.* 2024 [267]	Preclinical *in vivo* (mouse)	EAE	Attenuated severity
Hoyle *et al.* 2022 [268]	Preclinical *in vitro*	Macrophage - glial- neuron cultures	Decreased NLRP3 activation and IL1 production
Sangineto *et al.* 2024 [269]	Preclinical *in vitro*	Human microglia cultures	Decreased glycolysis, mitochondrial respiration modulation
Yu *et al.* 2024 [270]	Preclinical *in vivo* (mouse)	HFD mice	ITA ameliorates metabolic dysfunction
Pan *et al.* 2023 [271]	Preclinical *in vivo (mouse)*	HFD mice	ITA Improves cognitive function
Eberhart *et al.* 2024 [272]	Preclinical *in vivo*	HFD mice	ITA induces obesogenic changes in gut microbiota
Vázquez-Fresno *et al.* 2015 [273]	PREDIMED clinical trial subanalysis	Plasma biomarkers	Increased cisaconitate levels after MPD adoption
**Short Chain Fatty Acids (SCFAs)**			
He *et al.* 2021 [281]	Preclinical *in vitro*	CD8^+^ T Lymphocytes	Butyrate increased cytotoxic responses
Wang *et al.* 2024 [282]	Preclinical *in vitro*	CD8^+^ T Lymphocytes	Acetate inhibited cytotoxic responses
Nakamura *et al.* 2017 [283]	Preclinical *in vivo* (mouse)	Experimental uveitis	SCFAs decrease Th17 polarization
Nouri *et al.* 2014 [284]	Preclinical *in vivo*	EAE	EAE induction increases gut epithelial permeability
Haase *et al.* 2021 [285]	Preclinical *in vivo* Human biomarker	EAE MS patients	Propionic acid ameliorates gut permeability and EAE course Reduced fecal propionic acid, increased Th17 polarization
Duscha *et al.* 2020 [142]	Non-randomized clinical trial	MS patients	Propionic acid increases Treg numbers and reduces relapse rate and clinico-radiological progression
Caetano-Silva *et al.* 2023 [286]	Preclinical *in vitro*	Microglia	SCFAs reduce proinflammatory responses
Trend *et al.* 2021 [287]	Human Biomarker (cross-sectional)	MS patients	Reduced plasma propionate in MS
Park *et al.* 2019 [288]	Human Biomarker (cross-sectional)	Progressive MS patients	Reduced plasma acetate, butyrate and propionate
**α-ketoglutarate (AKG)/Glutamate/Glutamine**			
Wang *et al.* 2011 [292]	Preclinical *in vitro*	Murine T cells	MP upon activation increases glutaminolysis
Liu *et al.* 2017 [79]	Preclinical *in vitro*	Murine macrophages	AKG promotes M2 polarization upon LPS challenge
Matias *et al.* 2021 [293]	Preclinical *in vitro*	CD4^+^ T cells	AKG promotes Th1 polarization while sustaining OXPHOS
Xu *et al.* 2017 [76]	Preclinical *in vivo* (mouse)	T cell cultures EAE	Reduced AKG synthesis reduces Th17 polarization Reduced severity by GOT1-mediated AKG synthesis blockade
Magi *et al.* 2013 [298]	Preclinical *in vitro*	Neuron cultures	Glutamate through AKG anaplerosis sustains ATP production
Sulkowski *et al.* 2014 [300]	Preclinical *in vivo* (mouse)	EAE	Increased glutamate transporter expression
Ohgoh *et al.* 2002 [301]	Preclinical *in vivo* (mouse)	EAE	Altered Glutamate transporter expression (increased EAAC1, reduced GLAST, GLT-1)
Sarchielli *et al.* 2007 [303]	Preclinical *ex vivo*	Plasma from MS patients	Increased GLUR3 expression in patients with active disease
Ganor *et al.* 2003 [33]	Preclinical *ex vivo*	T cells from healthy controls Mouse EAE lymphocytes	Increased mGLUR3 mediated activation and chemotaxis
Fallarino *et al.* 2010 [304]	Preclinical *in vivo* (mouse)	EAE	mGLUR4 mediated signaling ameliorates EAE
Palmieri *et al.* 2017 [305]	Preclinical *in vivo* (mouse) – *in vitro*	EAE Microglia cultures	Loss of glutamine synthase in EAE Glutamine synthase decreases proinflammatory activation
Hollinger *et al.* 2019	Preclinical *in vivo* (mouse) – *in vitro*	EAE T cell cultures	glutaminase antagonist JHU-083 ameliorates severity JHU-083 inhibits T cell activation and proliferation
Kono *et al.* 2019	Preclinical *in vivo* (mouse)	EAE	Glutaminase blockade inhibits Th17 polarization and glycolysis
Kumar *et al.* 2022 [308]	Randomized cross-over clinical trial	Plasma and CSF from Mild cognitive impairment subjects and healthy controls	MPD improves CSF amyloid/tau pathology markers, neurofilament light chain levels and moduulates glutamate receptor expression
Al Gawwam *et al.* 2017 [309]	Human biomarker (cross-sectional)	Plasma from MS patients and controls	Increased plasma glutamate in MS patients
Sarchielli *et al.* 2003 [310]	Human biomarker (cross-sectional)	CSF from MS patients and controls	Increased CSF glutamate in MS vs controls, related to disease activity
Papandreou *et al.* 2020 [311]	PREDIMED clinical study subanalysis	Plasma biomarkers	Circulating glutamate unaffected by MPD adoption
**Fumarate (FA)**			
Hooftman *et al.* 2023 [315]	Preclinical *ex vivo*	Human macrophages	Fumarate accumulation linked to reduced IL10 secretion
Hammer *et al.* 2018 [316]	Preclinical *ex vivo* (longitudinal)	Lymphocytes from DMF treated MS patients	Increased Treg proliferation
Lima *et al.* 2024 [318]	Preclinical *in vivo* (mouse)	EAE	Dimethylfumarate increased Treg differentiation; disease severity amelioration
Kornberg *et al.* 2018 [319]	Preclinical *ex vivo* – *in vivo* (mouse)	Macrophages Lymphocytes EAE	Decreased activation by GAPDH inhibition Decreased glycolysis, decreased Th1/Th17 polarization increased treg Polarization GAPDH blockade ameliorates severity
Ntranos *et al.* 2019 [75]	Preclinical *ex vivo* (cross sectional)	Lymphocytes	Altered Th17 differentation by DMF mediated by epigenetic changes
Sarkar *et al.* 2022 [320]	Preclinical *ex vivo*	Mesenchymal stromal cells from MS patients	Decreased mitochondrial FH
Santos *et al.* 2023 [223]	PREDIMED clinical trial subanalysis	Plasma biomarkers	No variations in circulating FA after MPD intervention
**Ceramides**			
De Lira *et al.* 2020 [328]	Preclinical - *ex vivo*	Human T lymphocytes	neutral sphingomyelinases are needed to sustain prolonged T cell activation
Checa *et al.* 2017 [329]	Human biomarker (cross-sectional)	Plasma from patients with SLE	Increased C:16 and C:24 associated with disease activity
Miller *et al.* 2017 [330]	Preclinical *in vivo* – *In vitro*	EAE Oligodendrocyte Cultures	Ceramides tied to increased severity Ceramide synthesis blockade ameliorates neurotoxicity
Vidaurre *et al.* 2014 [331]	Preclinical *ex vivo*	Rat neuron cultures	C:16 and C:24 from MS CSF impair energy metabolism
Gil *et al.* 2020 [333]	Preclinical *in vitro*	Neuron cultures	Fingolimod reduces menadione-induced mitochondrial impairment
Van Doorn *et al.* 2012 [335]	Post-Mortem	Astrocyte cultures	Fingolimod reduces ceramide synthesis
Wang *et al.* 2017 [339]	PREDIMED clinical trial subanalysis	Plasma biomarkers	No significant changes in circulating ceramides after 1 year of MPD
Mathews *et al.* 2017 [340]	FRUVEDomic clinical trial subanalysis	Plasma biomarkers	Increased short-term C:16; decreased short-term C:24 after 8 weeks of vegetable enriched diet
Walker *et al.* 2019 [341]	Framingham offspring cohort study subanalysis	Plasma biomarkers	MPD adherence associated to decreased C:16

## LIST OF ABBREVIATIONS

2HG: 2-Hydroxyglutarate
α-KG: α-Ketoglutarate
ACL: ATP-citrate Lyase
ACOD1: cis-aconitate Decarboxylase
AD: Alzheimer’s Disease
AGE: Advanced Glycation End Products
ATF3: Activating Transcription Factor 3
BBB: Blood Brain Barrier
BDNF: Brain-derived Neurotrophic Factor
BTK: Bruton's Tyrosine Kinase
CA: Citrate
CIC: Citrate Carrier
CNS: Central Nervous System
CSF: Cerebrospinal Fluid
D2HG: R-2 Hydroxyglutarate
DAMPs: Damage Associated Membrane Patterns
DASH: Dietary Approach to Stop Hypertension
DHODH: Dihydroorotate Dehydrogenase
EAE: Experimental Autoimmune Encephalitis
EGCG: Epigallocatechin-gallate
FA: Ffumarate
FH: Fumarate Hydratase
GAPDH: Glyceraldehyde 3-phosphate Dehydrogenase
GPR: G-protein Coupled Receptors
GSK3-β: Glycogen Synthase Kinase 3-beta
HIF: Hypoxia Inducible Factors
HLA: Human Leukocyte Antigen
IDH: Isocitrate Dehydrogenase
IF: Intermittent Fasting
ITA: Itaconate
KD: Ketogenic Diet
L2HG: L-2 Hydroxyglutarate
LDH: Lactate Dehydrogenase
LPS: Lipopolysaccharide
MAPK: Mitogen-activated Protein Kinase
MP: Metabolic Programming
MPDs: Mediterranean Pattern Diets
MS: Multiple Sclerosis
mTOR: Target of Rapamycin
NFAT: Nuclear Factor of Activated T cells
NK: Natural Killer
NLRP3: NOD-like Receptor Protein 3
NRF2: Nuclear Factor Erythroid 2-related Factor 2
OXPHOS: Oxidative Phosphorylation
PD: Parkinson’s Disease
PDH: Pyruvate Dehydrogenase
PFK: Phosphofructokinase
PGC1-α: Peroxisome Proliferator-Activated Receptor Gamma Coactivator-1 alpha
PGDH: Phosphoglycerate Dehydrogenase
PI3K: Phosphatidylinositol3-kinase
PK: Pyruvate Kinase
PKC: Protein Kinase C
QoL: Quality of Life
ROS: Reactive Oxygen Species
RRMS: Relapsing Remitting Forms
SCFA: Short Chain Fatty Acid
SDH: Succinate Dehydrogenase
TCA: Tricarboxylic Acid Cycle
Th1: T Helper Type 1
Th17: T Helper Type 17
Th2: T Helper Type 2
Th9: T Helper Type 9
Treg: T Regulatory
WD: Western Diet
